# Radiotherapy combined with nano-biomaterials for cancer radio-immunotherapy

**DOI:** 10.1186/s12951-023-02152-2

**Published:** 2023-10-30

**Authors:** Qingrong Dong, Tingyu Xue, Haili Yan, Fang Liu, Ruixue Liu, Kun Zhang, Yu Chong, Jiangfeng Du, Hui Zhang

**Affiliations:** 1 Department of Medical Imaging, First Hospital of Shanxi Medical University, Intelligent Imaging Big Data and Functional Nano-Imaging Engineering Research Center of Shanxi Province, Taiyuan, 030001 People’s Republic of China; 2 College of Pharmacy, Shanxi Medical University, Jinzhong, 030619 People’s Republic of China; 3 State Key Laboratory of Radiation Medicine and Protection, School of Radiation Medicine and Protection School for Radiological and Interdisciplinary Sciences (RAD-X) Collaborative Innovation Center of Radiation Medicine of Jiangsu Higher Education Institutions, Soochow University, Suzhou, 215123 People’s Republic of China; 4 Collaborative Innovation Center for Molecular Imaging of Precision Medicine, Shanxi Medical University, Taiyuan, 030001 People’s Republic of China

**Keywords:** Radiotherapy, Immunotherapy, Nano-biomaterials, Radio-immunotherapy, Tumor

## Abstract

Radiotherapy (RT) plays an important role in tumor therapy due to its noninvasiveness and wide adaptation. In recent years, radiation therapy has been discovered to induce an anti-tumor immune response, which arouses widespread concern among scientists and clinicians. In this review, we highlight recent advances in the applications of nano-biomaterials for radiotherapy-activated immunotherapy. We first discuss the combination of different radiosensitizing nano-biomaterials and immune checkpoint inhibitors to enhance tumor immune response and improve radiotherapy efficacy. Subsequently, various nano-biomaterials-enabled tumor oxygenation strategies are introduced to alleviate the hypoxic tumor environment and amplify the immunomodulatory effect. With the aid of nano-vaccines and adjuvants, radiotherapy refreshes the host's immune system. Additionally, ionizing radiation responsive nano-biomaterials raise innate immunity-mediated anti-tumor immunity. At last, we summarize the rapid development of immune modulatable nano-biomaterials and discuss the key challenge in the development of nano-biomaterials for tumor radio-immunotherapy. Understanding the nano-biomaterials-assisted radio-immunotherapy will maximize the benefits of clinical radiotherapy and immunotherapy and facilitate the development of new combinational therapy modality.

**Graphical Abstract:**

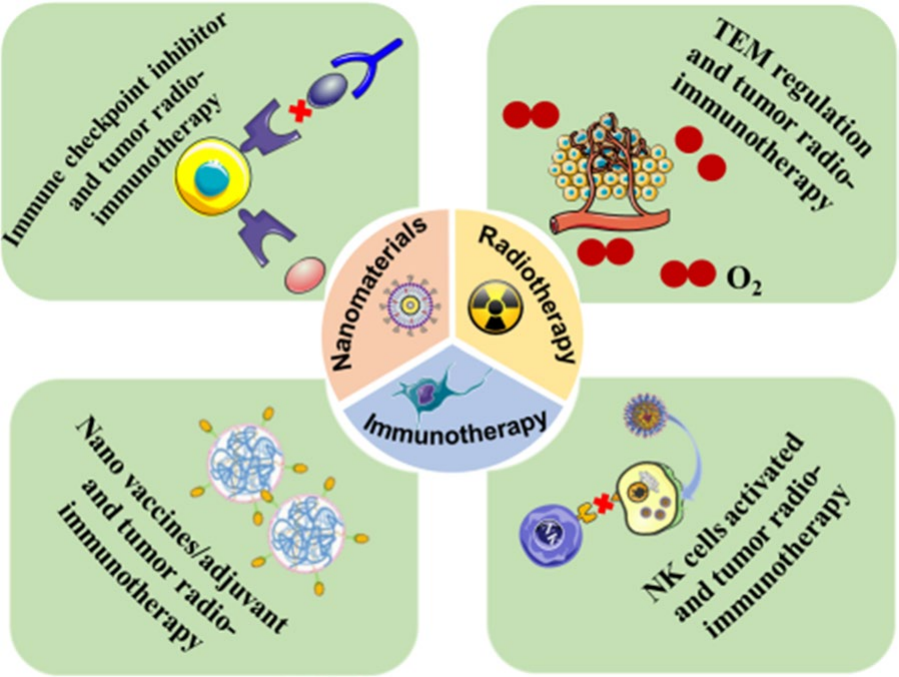

## Introduction

Radiotherapy, which mainly transfers energy to destroy the target tumor cells through ionizing radiation-induced oxidative stress and DNA double-strand breaks, remains a cornerstone in cancer treatment. According to statistics, radiotherapy plays an important role in more than half of patients with new-onset or recurring malignancies [1]. Although remarkable achievements have been received, it still has great limitation to acquire the satisfying radiotherapeutic efficacy toward recurrent and distal tumors [2, 3]. Meanwhile, ionizing radiation causes irreversible damage to the surrounding normal tissues, limiting the radiation dose delivered to patients [4]. With the booming development of biotechnology and nanotechnology in decades, varieties of functional nano-biomaterials have been applied in tumor treatment to achieve a better therapeutic effect at a lower irradiation dose and protect the surrounding normal tissues from radiation damage.

Remarkably, clinical studies have shown that large amounts of tumor-specific antigens released from irradiated tumor will be presented by antigen-presenting cells (APC) to cytotoxic CD8^+^ T cells, which can attack tumor cells far away from the radiation field [5, 6]. Radiotherapy-induced abscopal effect suggests that the local radiotherapy can not only directly eradicate in-situ cancer cells, but also produce a systemic immune response to cause immunogenic cell death [7, 8]. However, the anti-tumor immune response activated by ionizing radiation is insufficient to prevent metastatic and recurrent tumors. To enhance the abscopal effect caused by radiotherapy, concurrent tumor radiotherapy with immunotherapy is gradually developed in recent years [9]. Many clinical data show that combined immunotherapy and radiotherapy is effective in triggering tumor immunogenicity [10–15]. The Phase I/II trial enforced by Yuan et al*.* demonstrates the benefit and feasibility of anti-PD-1 (nivolumab) with high-dose-rate brachytherapy in patients with Grade Group 5 prostate cancer (PCa) [16]. Moreover, a recent retrospective analysis shows that patients treated by CTLA-4-targeting ipilimumab and radiotherapy had better clinical outcomes than those cared of ipilimumab alone [17]. Unfortunately, combination therapies involving immunotherapy and radiotherapy still have major challenges, including low response rate and therapies-related side effect. In addition, repeated radiation on tumor cells may induce chronic type I interferon and interferon-stimulated gene expression, thereby mediating radiation resistance and metastasis transmission through multiple inhibitory pathways [18]. Thus, it is vital to develop new combination therapy modalities or vehicles with low-toxicity and high-efficiency.

At present, a variety of functional nano-biomaterials have been used in radio-immunotherapy to achieve better therapeutic effect (Table 1) [19]. Among them, (i) High-Z materials (such as Au [20], Hf [21], Pt [22], Te [23] and Bi [24]), iron oxide nanoparticles [25], and MOF-based [21, 26–28] nano-biomaterials can work together with immune checkpoint inhibitors for radio-immunotherapy to enhance tumor immune response via strong X-Ray attenuation capabilities. (ii) MnO_2_ [29] and iron porphyrin-based nano-biomaterials [25] exert oxygen production function, which can not only adjust the hypoxic tumor microenvironment (TME), but also amplify the immunomodulatory effect. (iii) With the aid of nano-vaccines and adjuvants composed of immunogenic bacteria, viruses and biopolysaccharides, radio-immunotherapy has a good therapeutic effect on metastatic tumors and produces immune memory effect. (iv) Selenium-containing nano-biomaterials [30, 31] inhibit the expression of human leukocyte antigen E (HLA-E) on cancer cells to enhance the anti-tumor immunity mediated by NK cells, opening up a new approach for cancer radio-immunotherapy. In short, the introduction of functional nano-biomaterials not only exerts their intrinsic physicochemical performance, but also renews the host's innate or adaptive immune system, which has great application potential in cooperating with radiotherapy to assist tumor radio-immunotherapy.

**Table 1:** Represent nanomaterials for radio-immunotherapy

Radiotherapy type	Nano materials	With or without combine therapy	Sensitization principle	Tumor type
X-ray	AC-NPs	αPD-1	Captures tumor-derived protein antigens, transportes them to antigen-presenting cells	Melanoma metastasis [41]
X-ray	WSP NPs	αPD-L1	Increases the sensitivity of radiotherapy	Metastatic breast carcinoma [42]
^177^Lu	^177^Lu@Au NC	αPD-L1	Increases the sensitivity of radiotherapy and down-regulates the expression of PD-L1	Metastatic breast carcinoma and colorectal cancer [20]
X-ray	αPD-L1-LNP	αPD-L1	Improves targeting efficiency, induces up-regulation of PD-L1	Glioblastoma [44]
X-ray	FeWO_X_-PEG	αCTLA-4	Promotes the tumor-infiltration of various types of immune cells	Metastatic breast carcinoma [25]
X-ray	DBP-Hf nMOF	IDOi	Increases the sensitivity of radiotherapy	Metastatic breast carcinoma and colorectal cancer [26]
X-ray	4PI-Zn@CaCO_3_	IDOi	Reverses acidity-induced radioresistance, suppresses the production of Kyn, inhibits tryptophan (Trp) metabolism	Metastatic breast carcinoma [64]
^131^I	^131^I-Cat/CpG/ALG	αCTLA-4	Relieves the tumor hypoxia	Metastatic colorectal cancer [52]
X-ray	PLGA-R837@ Cat	αCTLA-4	Relieves the tumor hypoxia	Metastatic breast carcinoma [54]
^177^Lu	^177^Lu-APPs-PEG	Without	Relieves the tumor hypoxia, enhance the infiltration of cytotoxic T cells	Metastatic breast carcinoma [22]
X-ray	Hf-DBP-Fe	αPD-L1	Attenuates hypoxia, enhance the diffusion of (ROS)	Melanoma metastasis [21]
X-ray	ACF@MnO_2_	αPD-L1	Relieves the tumor hypoxia, inhibits HIF-1, down-regulates PD-L1	Metastatic breast carcinoma and colorectal cancer [29]
X-ray	Ag_2_S QDs, NO	αPD-L1	Increases the sensitivity of radiotherapy, relieves the tumor hypoxia	Metastatic colorectal cancer Melanoma metastasis [57]
X-ray	nMOF-CpGs	αPD-L1	Releases DAMPs and tumor antigens, delivers PAMPs	Metastatic colorectal cancer [71]
X-ray	E7_43–62_–PC7A	Without	Enhances immunity in a STING-dependent manner	Lung metastases [73]
X-ray	cowpea mosaic virus (CPMV)	Without	Turn an immunologically “cold” tumor into an immunologically “hot” tumor	Epithelial ovarian cancer [77]
X-ray	BNP-PC7A/CpG	Without	Captures cancer neoantigens, enhance their uptake in DCs	Melanoma Neuroblastom [78]
X-ray	IMD@Hf-DBP/αCD47	αPD-L1	Increases the sensitivity of radiotherapy, reverses immunosuppressed macrophages	Metastatic colorectal cancer [28]
X-ray	GLP-BiNP	Without	Increases the sensitivity of radiotherapy, activates dendritic cells	Metastatic breast carcinoma [24]
X-ray	ANPs	Without	Induces mature DC migrate to the tumor-draining lymph node and initiates the T-cell expansion	Metastatic breast carcinoma [79]
X-ray	Ch/γ-PGA NPs	Without	Reverses immunosuppressed macrophages, improves the maturation level of DC cells	Metastatic breast carcinoma [80]
X-ray	PSeR/DOX	Without	Enhances NK cells' function	Lung metastases [30]
ɣ-ray	Cyt-SeSe-Cyt	Without	Enhances NK cells' function	Lung metastases [31]

**Fig. 1:**
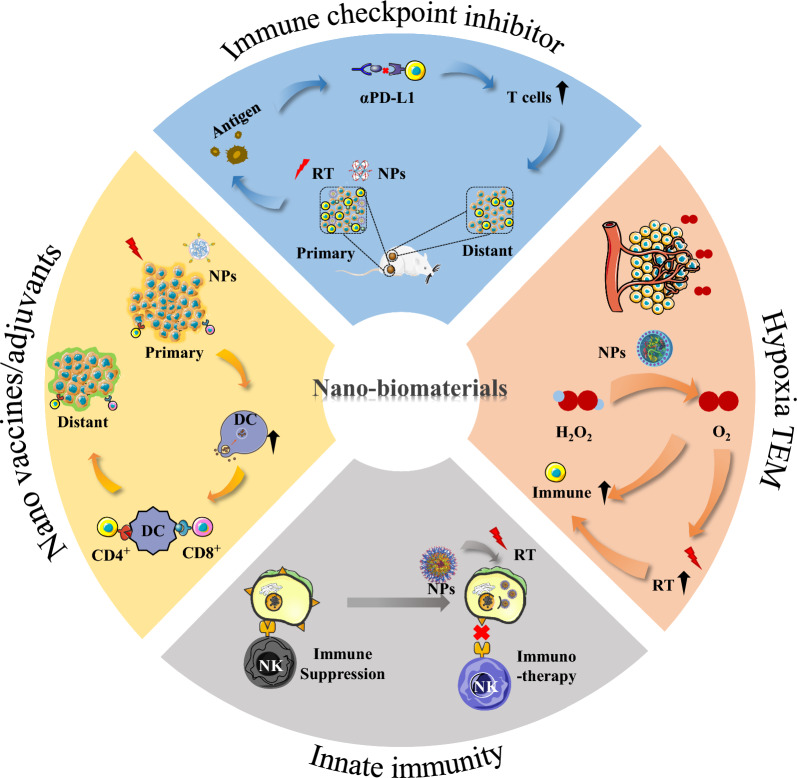
Schematic diagram of the main mechanism of radiotherapy combined with nano-biomaterials for cancer radio-immunotherapy

Therefore, in this review, we present the relevant research on the application of radio-immunotherapy with the assistance of functional nano-biomaterials in recent years (Fig. 1). We focus on the relationship between functional nano-biomaterials and radio-immunotherapy and expand on the following aspects, including (1) Nano-biomaterial-mediated radiotherapy combined with immune checkpoint inhibitors; (2) Nano-biomaterial-enabled regulation of the hypoxic and oxidation-stressed tumor microenvironment regulation for radio-immunotherapy; (3) Nano vaccines/adjuvants enhanced radio-immunotherapy; (4) Innate immunity activated by biological materials for radio-immunotherapy. Finally, we summarize the opportunities and challenges in the future development of nano-biomaterials-assisted radio-immunotherapy. We believe that our summary will facilitate the clinical translation of nano-biomaterials-assisted radio-immunotherapy and provide a guiding framework for clinicians and clinical researchers.

### Nano-biomaterial-mediated radiotherapy combined with immune checkpoint inhibitors

As the most promising treatment modality, tumor immunotherapy has been proven to be clinically effective in cancer fighting. In the midst of various immunotherapies, immune checkpoint blocking therapy, such as anti-Programmed Death 1 (αPD-1), anti-Programmed Death Ligand 1 (αPD-L1), anti-Cytotoxic T-Lymphocyte Antigen 4 (αCTLA-4), anti-Lymphocyte activation gene-3 (αLAG-3), anti-T cell immunoglobulin-3 (αTIM-3) and anti-T cell immunoreceptor with Ig and ITIM domains (αTIGIT), present successful therapeutic results in the treatment of many advanced malignancies by blocking the inhibitory pathways of immune cells [32–34]. Since the CTLA-4-targeting ipilimumab came on the market in 2013, the FDA has approved seven immune checkpoint inhibitors for the treatment of more than 20 cancer species [35]. Despite the encouraging success of immunotherapy in recent years, roughly 80% of patients are still insensitive to single-agent immune checkpoint blocking therapy [36, 37]. Additionally, the clinical use of immune checkpoint inhibitors is limited by grievous autoimmune-like adverse effects and secondary resistance [32, 38]. In this context, many preclinical and clinical research [12–14] suggest that radiotherapy is effective in promoting the activation and proliferation of tumor-specific cytotoxic T cells to improve the response rate of checkpoint blocking therapies [8]. For instance, studies have shown that the combination of RT + αTIM-3 significantly reduced tumor growth compared with single-agent αTIM-3 and RT [39]. In addition, in the patients with metastatic melanoma, the overall progression-free rate of combined radiotherapy with immune checkpoint inhibitors is about 36–50%, much higher than the sole treatment of immune checkpoint inhibitors [12, 14]. Although the survival benefit of the patient is improved, the therapeutic effect and response rates of combinational radio-immunotherapy is still limited, which is mainly affected by factors such as unsatisfactory radiotherapeutic efficacy and tumor metastasis. Fortunately, scientists have developed novel strategies by coupling multiple functionalized nano-biomaterials with radio-immunotherapy to further improve the therapeutic efficacy and immune response rate.

As we all know, the PD-1 receptor is expressed in activated CD8^+^ T cells. Once it binds to PD-L1 ligand in tumor cells, the function and proliferation of CD8^+^ T cells will be inhibited. Therefore, blocking the interaction of PD-1 with its ligands by αPD-1 or αPD-L1 is able to increase the effector CD8^+^ T cell activity in tumors [40]. Compared to other immune checkpoints, the PD-1 shows a very high expression level in the activated and lethal T cells. The introduction of nano-biomaterials in radiotherapy increases the amount of PD-1 by increasing the number of activated and lethal T cells, thereby improving the function of αPD-1 or αPD-L1 [27].

**Fig. 2:**
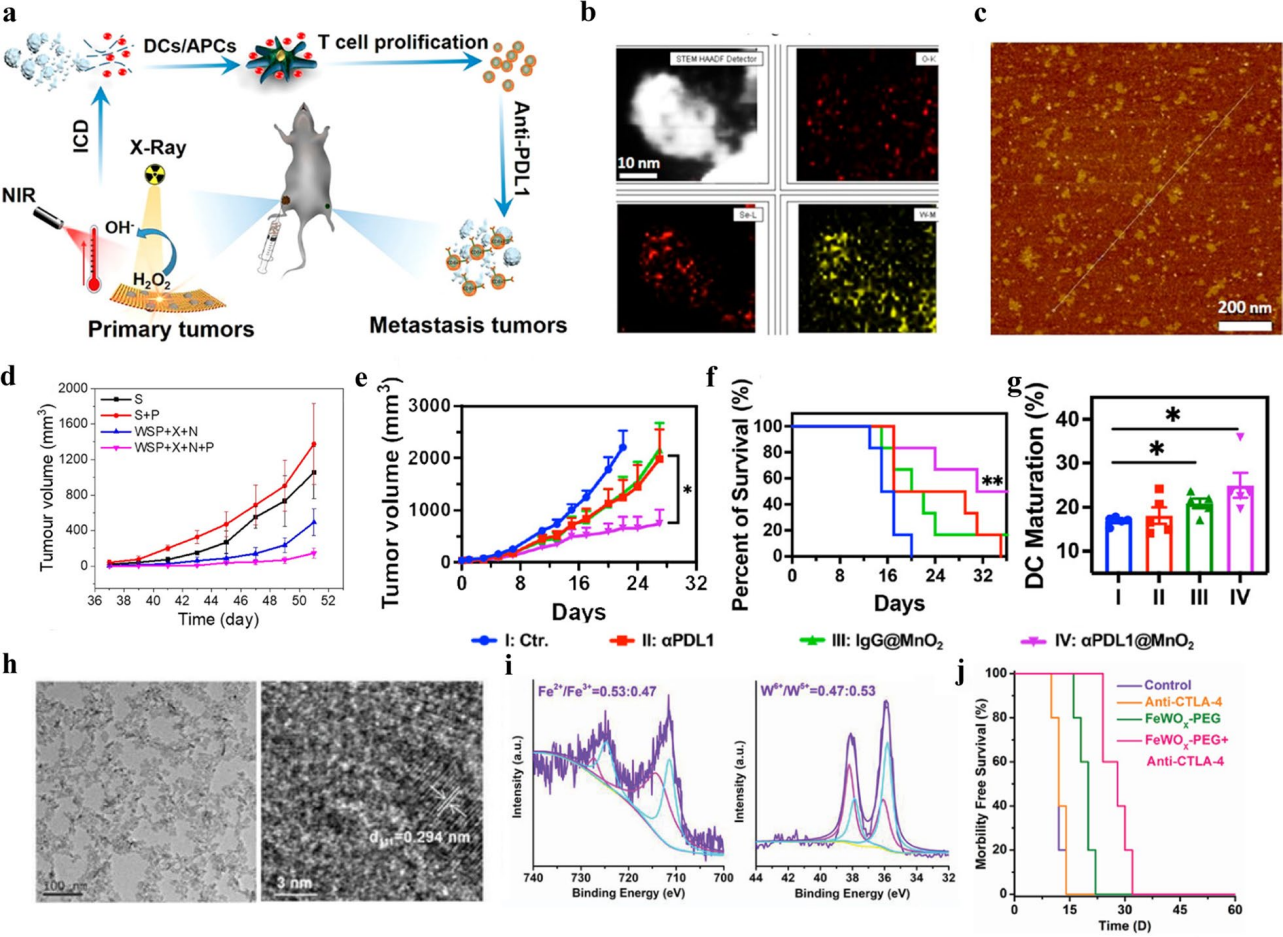
Nanomaterial-mediated radiotherapy combined with immune checkpoint inhibitors. **a** Schematic diagram of immune checkpoint blockade and abscopal effect of WO_2.9_-WSe_2_-PEG NPs-mediated RT. **b**, **c** Elements mappings image and AFM images of WO_2.9_-WSe_2_ NPs. **d** Tumors growth curves of mice model. Reproduced from ref 42. Copyright 2020, American Chemical Society. **e** Tumor growth curves and **f** survival rates of mice after different treatments. **g** Quantitative analysis of matured DCs after treatments. Reproduced from ref 43. Copyright 2023, American Chemical Society. **h** The magnified TEM image of FeWO_X_ NPs. **i** Fe 2p and W 4f XPS data of FeWO_X_ nanosheets in different binding-energy ranges. **j** The survival curves of mice model. Reproduced from ref 25. Copyright 2020, Wiley–VCH GmbH

Inspired by this idea, the combination of antigen capturing nano-biomaterials with αPD-1 or αPD-L1 to enlarge the X-ray-elicited immune response has been widely studied. For instance, Wang et al. [41] engineered several types of antigen capturing poly(lactic-co-glycolic acid) nanoparticles (AC-NPs) that could transport X-ray-stimulated tumor-specific proteins to antigen-presenting cells, which improves the efficacy of checkpoint inhibitors αPD-1 and induces the potent abscopal effect. With the addition of AC-NPs, the cure rate of distant tumor increased from 0 to 20%. In addition to delivering tumor-derived specific proteins, nano-biomaterials also could amplify the immunogenicity of radiotherapy through radiation sensitization. For instance, Dong et al*.* [42] designed semiconductor heterojunction structured WO_2.9_-WSe_2_-PEG nanoparticles (WSP NPs) with αPD-L1 antibody to ablate cancer. Upon X-ray irradiation, the WSP NPs combined with αPD-L1 contributed to a high regression of both primary tumor (> 90%) and distant tumors (> 80%) (Fig. 2a–d). In a different study, a Mn^2+^ chelated tannic acid-based nanoplatform was used to treat tumors by integrating αPD-L1. Compared to the αPD-L1-treated group, tumor tissue with combinational treatment showed onefold rise in survival rate (Fig. 2e–g) [43].

These results gave valid evidence that nano-biomaterials combined with X-ray irradiation could trigger CD8^+^ T cell infiltration and improve αPD-1 immunotherapy via enhancing the presentation of tumor-derived specific proteins or improving tumor radiosensitivity and immunogenicity.

In contrast to external radiation, internal radiotherapy with prolonged low-dose rate exposure, has also been reported to produce strong radio-immune effects and increase the αPD-L1 response rate. By labeling therapeutic radionuclide Lutecium-177 (^177^Lu) on the metabolizable gold nanoclusters (^177^Lu@Au NCs), Pei et al*.* [20] discovered that ^177^Lu@Au NCs could not only effectively stimulate the maturation of dentritic cells (DCs), but also promote the expression of PD-L1 on remote tumor, which enhanced the probability of αPD-L1 binding to the tumor. Subsequently, the effectiveness of this strategy was verified in transgenic mice with spontaneously metastatic tumors. It was found that the combination of ^177^Lu@Au NCs and αPD-L1 effectively inhibited tumor growth and metastasis, and prolonged the survival cycle of mice.

Notably, downregulating the PD-L1 ligands or eradicating PD-L1^+^ cells also reduce the immune escape of tumor cells, thereby enhancing the anti-tumor immune response. For instance, Zhang et al*.* [44] designed PD-L1-targeted lipid nano-biomaterials (LNP) co-loaded with αPD-L1 and the cyclin-dependent kinase inhibitor dinaciclib for glioblastoma radio-immunotherapy. Under X-ray radiation, the delivery efficiency of the sanatory payload was greatly enhanced owing to the up-regulation of PD-L1 on tumor-associated myeloid cells. As a result, the treatment of αPD-L1-LNP/Dinaciclib not only inhibits the function of PD-L1, but also restrains the de novo synthesis of PD-L1. Ultimately, tumor-associated myeloid cells were eliminated by αPD-L1-LNP and the survival of mice was greatly extended when combined with radiation therapy. Similarly, Erel-Akbaba et al*.* [45] produced tumor-targeting solid lipid nanoparticles (SLN) to deliver small interfering RNAs against glioblastoma. With the low-dose radiation increased uptake of SLN into the brain tumor region, the resulting nano-biomaterials down-regulated the expression of tumor PD-L1 and increased the median survival of mice from 21 to 38 days.

CTLA-4 is another crucial immune checkpoint expressed most heavily on regulatory T cells (Treg) and activated T cells. When CTLA-4 binds to CD80 or CD86, it leads to the transcytosis of the ligands on antigen-presenting cells (APC), resulting in reduced levels of ligands on the APC surface. The immune escape of tumor cells could be cut off by αCTLA-4, which increases the proliferation and function of cytotoxic T cells. It has been known that the local reactive oxygen species (ROS)-induced inflammation could elicit strong immune response but simultaneously activate immune suppressive Treg cells [46, 47]. Hence, with the increased ROS generation by nano-biomaterials-sensitized radiotherapy, the introduction of αCTLA-4 is capable of amplifying the anti-tumor immune responses. For instance, Gong et al*.* [25]described that FeWO_X_ nanosheets heighten anticancer efficacy by coupling with radiotherapy and CTLA-4 checkpoint blockade. The FeWO_X_ nanosheets significantly improve cancer radiotherapy by the depletion of endogenous glutathione and amplification of ROS generation. Then oxidative stress-induced inflammation triggers robust immune responses, which is further amplified by CTLA-4 checkpoint blockade. It was discovered that Treg cells of the combination therapy group were obviously decreased. Eventually, the treatment of radiated FeWO_X_-PEG plus αCTLA-4 showed the most significant prolonged survival time of mice and greatly inhibited the tumor growth of distant tumors (Fig. 2h–j).

In a word, nano-biomaterials combined with radiotherapy can be used in immunotherapy with checkpoint inhibitors to break immune tolerance and improve overall response rate by the activation of cytotoxic T cells, which show great advantages in tumor growth and metastasis inhibition. However, there is currently limited research on the combination of radiotherapy and nano-biomaterials for other immune checkpoints (except PD-1 and CTLA-4), Therefore, whether the remarkable therapeutic responses observed with PD-1 and CTLA-4 can be reproduced in other immune checkpoints remains to be determined in future studies.

### Nano-biomaterial-enabled regulation of the hypoxic and oxidation-stressed tumor microenvironment regulation for radio-immunotherapy

Hypoxia is an important hallmark of solid TME, which has a far-reaching negative influence on the anticancer effect of immunotherapy [48]. It has been known that hypoxic TME results in the infiltration of huge amounts of M2-polarized macrophages at the site of the tumor, reduces lymphocyte accumulation, suppresses infiltrating effector cells, and improves the activity of hypoxia-inducible factor (HIF), thereby leading to the immunosuppression of tumor tissues [49]. In addition, the efficacy of radiotherapy is greatly compromised by the hypoxic microenvironment owing to the decreased radiation-induced DNA damages and generated therapeutic resistance [50]. Therefore, modulating the tumor hypoxic conditions is of great clinical value in improving radiotherapy efficiency and reversing immunosuppression [51]. Recently, many researchers have developed numerous tumor oxygenation strategies to improve the efficiency of radiotherapy and strengthen immunotherapy outcomes. Catalase is a natural enzyme to trigger the dissolution of excess H_2_O_2_ to generate O_2_. Inspired by this feature, Chao et al*.* [52]investigated a sodium alginate (ALG) formulation incorporating catalase (Cat) merged into the therapeutic ^131^I radioisotope (^131^I-Cat/ALG) to relieve tumor hypoxia for cancer treatment. The researchers found an improved survival rate in the ^131^I-Cat/ALG group (survived for over 60 days) compared with the other groups (survived for 16 ~ 24 days). Therefore, the results show that catalase could be trapped in the tumor cell by ALG hydrogel for a long time to ameliorate hypoxic state, broke through the dependence of internal radiotherapy on oxygen, optimized internal radiotherapy efficacy, and eliminate local solid tumors. At the same time, the improvement of intratumor hypoxia could also enhance the infiltration rate of immune cells and improve the effect of immunotherapy. The nano-biomaterials combined with αCTLA-4 amplified the anti-tumor immunity effect of αCTLA-4, and the metastatic tumors were basically eliminated.

Moreover, recent studies have indicated that several functional nano-biomaterials could reactivate immunosuppression in the tumor and enhance the tumor sensitivity to radiation immunotherapy via reversing the hypoxic state in TME and promoting infiltration of various types of immune cells [53]. Chen et al*.* [54] indicated that a core–shell nanoparticles based poly (lactic-co-glycolic) acid (PLGA) loading catalase and hydrophobic imiquimod (R837) (PLGA-R837@Cat) combined with external radiotherapy showed the same ability to strengthen immune stimulation. After injecting PLGA-R837@Cat nanoparticles, a significant reduction of M2-polarized macrophages was observed in the tumor. Suggested that the tumor microenvironment changed from immunosuppressive to immunostimulant. Compared with PLGA-R837 for mice postradiotherapy, PLGA-R837@Cat combined with RT showed a higher percentage of DCs maturation, which mainly verified the existence of catalase could relieve tumor hypoxia, promote radiation-caused tumor breaking, and produce more tumor-associated antigens (TAAs) to heighten immune stimulation.

**Fig. 3:**
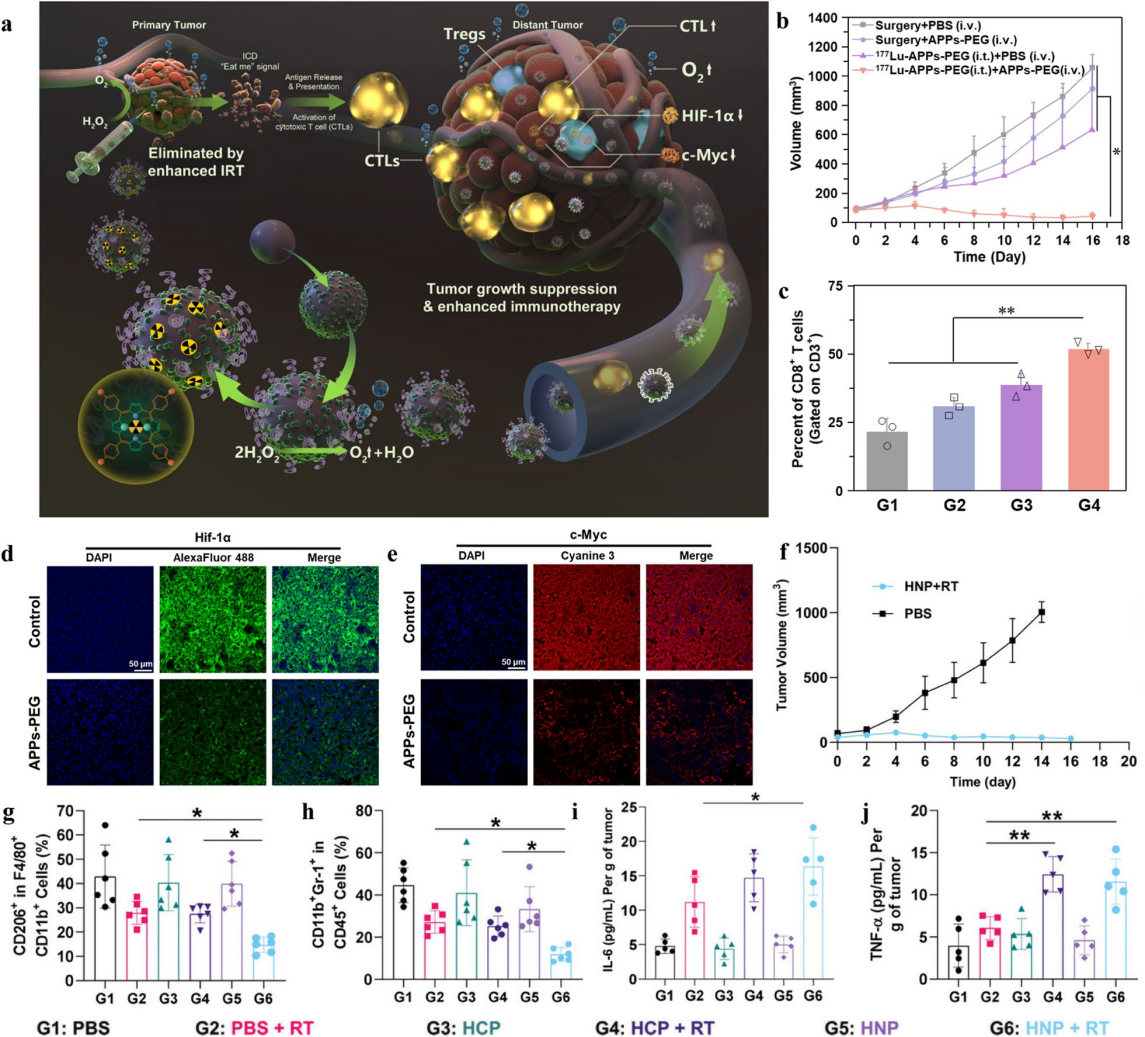
Nano-biomaterial-enabled regulation of the hypoxic and oxidation-stressed tumor microenvironment regulation for radio-immunotherapy. **a** Schematic illustration of enhancing anti-tumor immunotherapy by radioactive nano-oxygen generator. **b** Distant tumor growth curves of mice model. **c** The percentage of tumor-infltrating CD8^+^ T cells (G1: PBS + Surgery, G2: APPs-PEG + Surgery, G3: PBS + ^177^Lu-APPs-PEG, G4: ^177^Lu-APPs-PEG + APPs-PEG). **d**–**f** The immunofluorescence slices showing the expression of HIF-1α (**d**), c-Myc (**e**) and Ki67 (**f**) in tumors. Reproduced from ref 22. Copyright 2021, Elsevier Ltd. **g** M2-like macrophages (CD11b^+^F4/80^+^CD206^+^). **h** MDSCs (CD45^+^CD11b + Gr-1^+^). **i**, **j** The levels of IL-6 (**i**) and TNF-α (**j**) in tumors with different treatments as indicated. Reproduced from ref 59. Copyright 2023, Wiley–VCH GmbH

Noteworthily, the discovery of nanozyme instead of nano-vector loaded catalase could further simplify the operation process and reduce the cell toxicity. Recent studies have shown that in addition to enhancing radiotherapy, improving the hypoxic TME could directly advance the anti-tumor immune response by increasing the infiltration of immune cells at the tumor site. Herein, Pei et al*.* [22] established a nano-oxygen generator composed of ^177^Lu labelling metal–organic framework (MOF) with in-situ grown Au-Pt nanozyme (^177^Lu-APPs-PEG) (Fig. 3a). APPs-PEG treatment group (^177^Lu-APPs-PEG eliminated tumors in situ and further assisted by APPs-PEG) has significantly increased the ratio of cytotoxic T cells (up-regulated to 51.93 ± 2.25%) and inhibited distant tumors, ultimately extending the survival time of four-fifths mice (Fig. 3b, c). As summarized, the increase of O_2_ in distal tumors further improved the infiltration of cytotoxic T cells into the solid tumors and meanwhile promoted the anti-tumor immune reaction (Fig. 3d–f).

In addition to Au-Pt nanozyme, other nano-biomaterials, such as Fe based nanozyme, Mn based nanozyme, are also reported as a catalase-like nanozyme to dissolve H_2_O_2_ into oxygen molecules. For instance, Ni et al*.* [21] reported a biomimetic nanoscale metal–organic-framework (nMOF) with αPD-L1 for synergistic antitumor. The Hf-DBP-Fe unit in nMOF structure could decompose the excessive H_2_O_2_ inside the tumor to generate O_2_ and hydroxyl radical (•OH). 83.33% of inhibition in both primary and distant tumors growth was achieved after Hf-DBP-Fe ( +)/αPD-L1/RT treatment. This study immensely improved the treatment of local tumors by ionizing radiation and induced systemic antitumor immunity in a non-T cell inflammatory tumor phenotype.

In the case of hypoxia in the tumor microenvironment, HIF-1α protein, the master regulator of tumorigenesis and mitochondrial respiration, cannot be recognized and degraded, leading to infinite proliferation of tumor cells. The previous studies have shown that the tumor oxygenation strategy cannot effectively inhibit the expression of HIF-1α, which induces tumor resistance to radiation [55]. Therefore, residual HIF-1 functional inhibition combined with tumor oxygenation is able further optimize therapeutic outcomes of radio-immunotherapy [56]. Meng et al*.* [29] designed a ROS responsive nanoplatform that successfully coated cationic acridine yellow and some hydrophilic cationic drugs into MnO_2_ nanoparticles (ACF@MnO_2_). It was found that 78.90% of inhibition in distant tumors after ACF@MnO_2_ plus radiotherapy treatment. In contrast, after αPD-L1 treatment, the inhibition of mice was 67.59%. These results indicated that ACF@MnO_2_ has the effect of inhibiting HIF-1α/β dimerization and the potential to substitute for checkpoint inhibitors.

In addition to increasing the amount of oxygen at tumor site, developing sensitizers that are effective against hypoxic tumors are also critical. Recent research shows that NO makes many immune cells, including T lymphocytes, macrophages, and antigen-presenting cells, active and against cancer. Therefore, Zhou et al*.* [57] developed a NO delivery system that is generated and released from the Ag_2_S QDs under the NIR irradiation, the Ag_2_S@BSA‑SNO combined with laser and X ray inhibited the tumor growth and survival rate of mice in combined treatment was 60% in at least 30 days. In another research, using sodium nitroprusside as a NO donor, a ^32^P-labelled single-layer 2D nanosheet, ZnNO(^32^P), was created by chelating both Zn ions and ^32^P radioisotopes. The therapeutic radioisotope ^32^P with β-ray emission effectively activated water and produced intense Cerenkov luminescence, which prompted NO release. The hypoxia-mediated immunosuppressive tumor environment was greatly improved by this long-term NO-releasing technique. Therefore, tumors treated with ZnNO(^32^P) exhibited the smallest Treg and HIF-1α positive areas and the highest CD8-positive cytotoxic T lymphocytes positive area. Moreover, the ZnNO(^32^P) and αPD-1 combination therapy induced a strong and long-lasting immunological response [58]. Furthermore, Liu et al*.* synthesized Hf-nIm@PEG nano-biomaterials to deposit radiation energy, trigger the release of NO, modulate the TME, and finally realize synergistic radio-immunotherapy (Fig. 3g–j) [59].

Moreover, the effectiveness of RT and immunotherapy is limited by the antioxidant system of TME. The primary reducing agent of cancer cells' antioxidant system, glutathione (GSH), can remove reactive oxygen species (ROS) and lessen the effectiveness of ROS-based treatments. In order to effectively break the antioxidant barrier in TME, Yu et al*.* developed Si-Mn-based NPs (SM NPs) by coating MnO_2_ onto SiO_2_ NPs, which not only causes ROS production but also depletes GSH in the tumor. In Lewis lung cancer cells, the SM NPs raised lipid peroxidation levels and effectively induced an increase in the number of cytokines secreted by macrophage-like cells, suggesting that it has a modulation function in immune responses [60].

Overall, the strategy of combining tumor radio-immunotherapy based on improving tumor microenvironment with nanotechnology is effective and has enormous potential for clinical translation. Catalase-delivered nanoplatform, nanozyme (including Au, Pt, Fe, and MnO_2_), and radiosensitizers have achieved some success in improving tumor hypoxia and built up the efficiency of radio-immunotherapy. Additionally, many researchers also designed corresponding nano-biomaterials to enhance the immune effect of radiotherapy based on express high levels of nutrients depleting related ectoenzymes (e.g., indoleamine 2,3-dioxygenase 1 (IDO-1) [61–64] in TME, and some reports focused on the physical tumor microenvironment [65].

### Nano vaccines/adjuvants enhanced radio-immunotherapy

Vaccine therapy could enhance peripheral tumor-specific T cell response and activate the innate immune system. Unfortunately, classical cancer vaccines confront several major challenges, including low efficiency of vaccine release, ineffective cross-presentation of tumor tissue-specific antigens, and restricted transmission of tumor antigens to lymph nodes because of enzymatic degradation and fast renal clearance [66–69]. It has been known that radiation therapy induces immunogenic cell death to generate tumor antigens and improves antigen presentation efficiency. Clinical studies [70] have demonstrated that proton-beam radiotherapy combined with in situ vaccine is safe and feasible for patients with hepatocellular carcinoma. The first clinical trial conducted by Abei et al*.* presented the effectiveness of the "in situ vaccination" strategy. Nine patients had a median progression-free survival of 6.0 months (range: 2.1–14.2), and four of them had a progression-free survival of more than a year [18]. Moreover, the nano vaccines prepared with nano-biomaterial vehicles enhance antigen uptake in DCs following radiotherapy, which facilitates antigen cross-presentation and motivates the antitumor T-cell response.

Inspired by this feature, Ni et al*.* [71] realized locally activable immunotherapeutic strategy using nanoscale metal–organic frameworks (nMOFs) loaded with CpG oligonucleotides, which has been widely explored as vaccine adjuvants for DCs maturation and pro-inflammatory cytokines secretion. Under X-ray radiation, high levels of ROS were generated to activate the release of DAMPs and tumor antigens. Meanwhile, the adhesion of antigen-presenting cells was promoted by delivered CpG oligonucleotides and the combined therapeutic strategy enlarged cytotoxic T cells in tumor-draining lymph nodes. Ultimately, both the primary and distant tumors were significantly subsided and this combination treatment generated a strong immune memory effect. In addition to CpG oligodeoxynucleotides, other immune adjuvants, such as stimulator of interferon (IFN) genes (STING) agonist, have also been developed as nanovaccines to combine with ionizing radiation for improving the cancer immunotherapy [72]. For instance, Luo et al*.* [73]developed an antigen-loaded polymeric PC7A NPs as STING-activating nanovaccines to boost systemic cancer-specific T cell response. When combined with X-ray radiation, local STING pathway was further activated and produced a synergistic treatment result against large tumors compared to sole treatment, which not only defeated the primary tumor but also observed an abscopal effect.

**Fig. 4:**
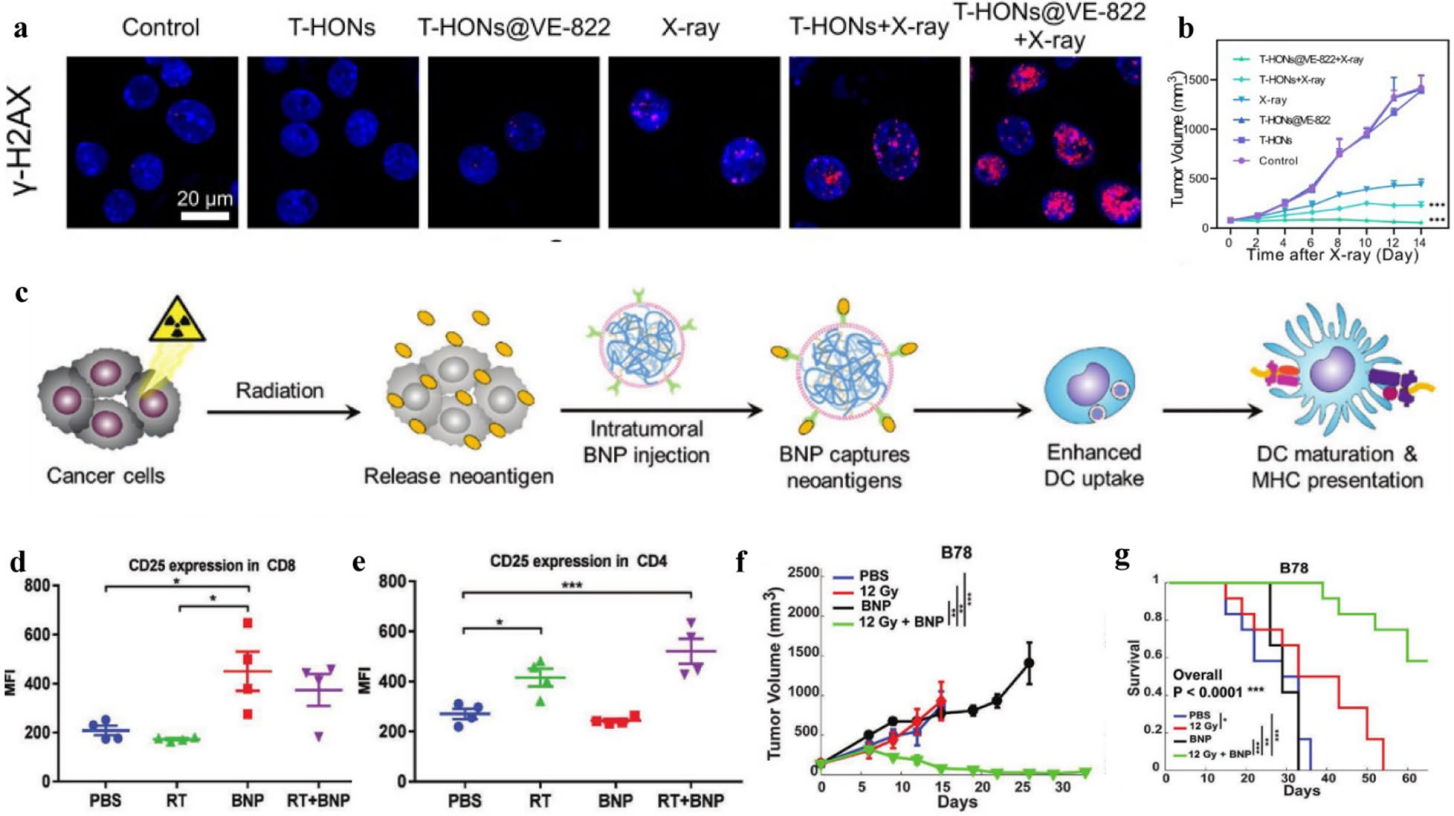
Nano vaccines/adjuvants and tumor radio-immunotherapy. **a** Images of DSBs tested by γ-H2AX foci. **b** Tumor volume growth curves of mice. Reproduced from ref 74. Copyright 2023, Elsevier B.V. **c** Schematic diagram of RT + BNP to enhance APC uptake and activation. **d**, **e** Expression levels of CD25 in tumors with different treatments and analyzed by flow cytometry and gene expression. The tumor volume (**f**) and growth rates analysis (**g**) in the B78 melanoma model. Reproduced from ref 78. Copyright 2019, Wiley–VCH Verlag GmbH & Co. KGaA, Weinheim

ATR kinase inhibitors (ATRi) not only prevent DNA damage repair but also act as an adjuvant to modulate the immune system and stimulate a more potent immunological response. Liu et al*.* created a nanocomposite consisting of hafnium oxide (HfO_2_) NPs and a hydrophobic ATRi VE-822 (Berzosertib). As radiosensitizers, HfO_2_ NPs could cause substantial harm to tumor cells when exposed to external X-ray radiation. After that, the loading VE-822 inhibits ATR and stops DNA damage repair, significantly enhancing the local radiotherapeutic efficacy (Fig. 4a). Importantly, the combination of VE-822 and HfO_2_ NPs-mediated RT can effectively stimulate the body's immune system to fight cancer by boosting immunogenicity based on the cGAS-STING pathway and promoting immune cell infiltration (Fig. 4b) [74].

Recently, the preparation of nano vaccines using immunogenic viruses and bacteria [75, 76] has also become a hot research topic in recent years. For instance, Patel et al*.* [77] prepared cowpea mosaic virus into nano-biomaterials for enhanced radio-immunotherapy. Patel et al*.* [78] used bacterial membranes and imine groups coated with PC7A/CpG to make nano vaccines (Fig. 4c). These nanoparticles could be combined with radiotherapy to transform immune “cold tumors” (low number of TILs) into immune “hot tumors”, with significantly reduced tumor metastasis rate and improved survival in treated mice (Fig. 4d–g).

Due to the high efficiency and low toxicity of biological polysaccharides, the substitution of TLR agonist by biological polysaccharide with specific immune function has also become a research hotspot. Yu et al*.* [24] prepared ganoderma lucidum polysaccharide doped bismuth sulfide to achieve dual effects of radiation sensitization and DCs activation. Subsequently, the astragalus polysaccharide nanoparticles (ANPs) designed by Peng et al*.* [79] enhanced the effect of radiotherapy-induced the maturation of DCs, further induced the migration of mature DCs to tumor-draining lymph nodes, and initiated T cell expansion. Compared with the PBS group, RT + ANPs increased the percentage of CD4^+^ T cells in the tumor-draining lymph nodes by nearly 20%. Recently, nano-biomaterials with innate immune activity are discovered to trigger systemic immunotherapy without the additional immune stimulants, further improving the safety of anti-tumor immunity. Castro et al*.* [80] reported that chitosan/poly(γ-glutamic acid) nanoparticles could reverse immunosuppressed macrophages, improve the maturation level of DCs.

In addition, CO‐based therapy has emerged as an immune vaccine to participate in anti-tumor immunity. For instance, Li et al*.* designed a nanovaccine by combining X-ray-triggered CO releasing lanthanide scintillator nanoparticles (ScNPs: NaLuF4: Gd, Tb@NaLuF4) with photo-responsive CO releasing moiety (PhotoCORM) for synergistic CO gas/immuno-therapy of tumors, which can be activated by external X-rays, and subsequently achieves the release of CO gas in deep tissues, along with Co-mediated ROS production and ICD. Therefore, nano-biomaterial-based cancer vaccines united with radiotherapy is a hopeful approach to stimulate the antigen presentation and activate peripheral tumor-specific T cell response [81].

These studies suggest that nano-biomaterials-based immunoadjuvant combined with radiotherapy could improve susceptibility to immunotherapy.

### Innate immunity activated by biological materials for radio-immunotherapy

Innate immunity involves various types of cells of the myeloid lineage, including DCs, macrophages, monocytes, mast cells, polymorphonuclear cells, and innate lymphoid cells (such as NK cells). Based on the powerful functions of innate immune cells such as recognizing tumors, regulating adaptive immunity, and killing tumor cells, many studies have manipulated innate immune cells to lyse malignant cells [82]. Radiotherapy is increasingly investigated to function as an immunomodulatory adjuvant to improve innate immunotherapy efficiency. For example, ionizing radiation can drive macrophages to differentiate into pro-inflammatory phenotypes with anti-tumor functions [83]. Considering the limited success of the combination therapy, considerable efforts have been devoted to combining nano-biomaterials with radiotherapy and immunotherapy to augment the anti-tumor immune responses.

**Fig. 5:**
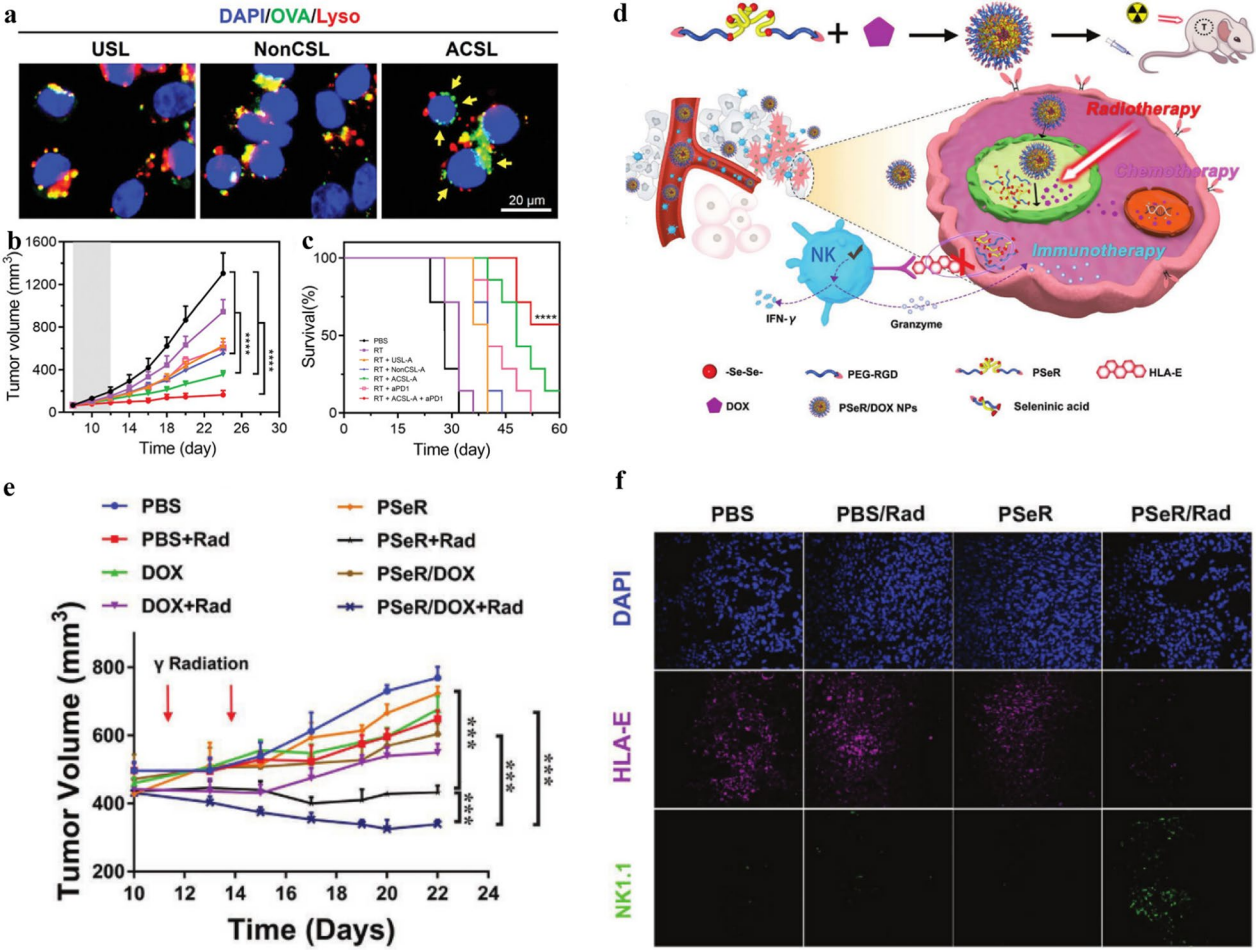
Innate immunity activated by biological materials for radioimmunotherapy. **a** CLSM imaging of the lysosomal escape of ovalbumin (OVA, green) in the presence of USLs, NonCSLs, and ACSLs in DCs after incubation for 12 h. The cells were stained with lyso-tracker (red). Scale bar, 20 µm. **b** Average tumor growth kinetics. **c** Survival of the mice. Reproduced from ref 85. Copyright 2021, Wiley–VCH GmbH. **d** Schematic diagram of combined chemotherapy, radiotherapy and immunotherapy using selenium nanoparticles. **e** Tumor volume of mice model after different treatments. **f** Confocal microscopy showing the tumor infiltration level of HLA-E (purple) and NK1.1(green). Reproduced from ref 30. Copyright 2020, Wiley–VCH Verlag GmbH & Co. KGaA, Weinheim

After radiotherapy, DCs would engulf TAAs which derived from tumor cells and process them into peptides, which are presented to major histocompatibility complex (MHC) on the cell surface. The MHC-antigen complexes could then be recognized by T cell receptors to activate T cells, triggering the subsequent immune responses [84]. However, TAAs production are limited after radiotherapy and most of TAAs internalized by DCs are degraded in lysosomes, resulting in suboptimal antigen cross-presentation. Nano-biomaterials can enhance radio-immunotherapy by increasing TAAs production, promoting TAAs lysosome escape, and further promoting cross-presentation. For example, a synthetic antigen-capturing stapled liposome, comprising liposomes, N,N′-methylenebis(acrylamide), 2-(hexamethyleneimino) ethyl methacrylate (C7A-MA), maleimide (Mal) and _L_-arginine, has been shown to increase the generation of TAAs, facilitate lysosomal escape and cross-presentation of TAAs in vitro (Fig. 5a). Furthermore, its combination with RT promoted an extended survival time. Seventy-five percent of the mice survived over 45 days, while all the mice in other treated groups were dead (Fig. 5b, c) [85].

Tumor-associated macrophages (TAMs) mainly play the role of endocytosis, processing and antigen presentation, which are classified mainly into the classically activated tumoricidal M1 phenotype and the tumor-supportive M2 phenotype [86]. Therefore, efforts have been underway to strengthen the tumor-negative function of macrophages through reeducating them from M2 phenotype toward M1 phenotype [87]. For example, Cao et al*.* reported CpG decorated gold (Au) nanoparticles to enhance improve radio-immunotherapy efficacy. In this study, Au NPs served as radioenhancers increased antigen production and CpG re-educated M2 TAMs to M1 TAMs, thus arousing innate immunity and meanwhile priming T cell activation. When the nanoparticles were combined with RT, a significantly increase and drop was observed in the expression of M1 and M2 cells, respectively [88]. In addition, Ni et al*.* [28] focused on the HF-DBA MOF loaded with IMD αCD47 and combine with αPD-L1 to treat bilateral colorectal tumors in mice. In this study, the platform could not only enhance X-ray energy deposition, generate a variety of ROS to enhance the function in antigen presentation, but also repolarize immunosuppressive M2 macrophages to immunostimulatory M1 macrophages, and completely eradicated distant tumors.

Nowadays, NK cells-based therapies have been emerging as a prominent tumor immunotherapy treatment in some cancers owing to their dual functions of cytotoxicity and immune regulation in eliminating tumor cells [89]. However, as human leukocyte antigen E (HLA-E) can inhibit the activity of NK cells, the overexpressed of HLA-E within tumor protects tumor cells of various origins from lysis by natural NK cells [90]. As human leukocyte antigen E (HLA-E) inhibits the activity of NK cells, tumors cannot be destroyed by natural NK cells through overexpression of HLA-E [67]. Therefore, the NK cells are unable to effectively clear tumor cells.

A recent study proved that selenic acid could block HLA-E expression and facilitate cellular apoptosis in tumor cells, which evokes NK cells-mediated antitumor activity. However, the systemic administration of selenic acid possibly raises cytotoxicity of NK cells to HLA-E-expressing normal cells. Therefore, selenic acid in situ generated within tumor site is of great significance in NK cells-based therapies. It has been reported that diselenide bonds could be cleaved by ionizing radiation and transformed into seleninic acid. As a result, nano-biomaterials with radiation-sensitive diselenide bonds can be designed to receive the NK cells-activated tumor therapy and reduce the damage of NK cells to normal tissues.

For this purpose, Gao et al*.* [30] developed a nanomedicine (PSeR/DOX) that includes an X-ray-sensitive diselenide-based polymer skeleton and chemotherapeutic agent doxorubicin (DOX). Under ionizing radiation, diselenide bonds could be cleaved and oxidized into selenic acid, which enhanced NK cells-mediated cytotoxicity (Fig. 5d). When integrated with X-ray, mice treated with PSeR/DOX observably increased the tumor infiltration of NK1.1( +) NK cells, abated the expression level of HLA-E, and promoted the tumor inhibition rate, which was linked to the incorporation of radiotherapy, selenic acid-mediated immunotherapy, and chemotherapy (Fig. 5e, f). Apart from X-rays, γ-rays, which are more penetrating, are also used to break diselenide bonds. Li et al*.* [31] co-assembled pemetrexed nano-biomaterials between cytosine disselenides and pemetrexed nano-biomaterials by γ-radiation-sensitive hydrogen bonding, enhancing the sensitivity of cancer cells to NK cells and significantly inhibiting tumor metastasis.

These ionizing radiation-responsive nano-biomaterials, through radiation in situ release of payloads, achieve comprehensive treatment for malignant tumors. Nano-biomaterials responsive to ionizing radiation provide a new strategy for immunotherapy controlled by ionizing radiation and expand the application field of radiotherapy, but its practical application and clinical transformation need to be further explored. The internal mechanism between radiation dose and material sensitivity needs to be further studied, although radiation may improve controllable penetration.

## Conclusions and perspectives

In recent years, immunomodulatory benefits of radio-immunotherapy, such as inducing ICD, leading to the release of DAMPs, and enhancing tumor immunogenicity, have been widely explored. To improve the response rate of combined immunotherapy with radiation therapy, varieties of nano-biomaterials or nanomedicines were designed for nano-biomaterials-assisted radio-immunotherapy. For instance, the combination of high Z nanoradiosensitizers under X-ray irradiation and immune checkpoint blockades can favor anti-tumor immunities. Meanwhile, the radio-immunotherapy efficacy is enhanced by oxygen-producing nanomedicines for the adjustment of the immunosuppressive TME, which enhances their anti-cancer efficacy. In addition, novel nano vaccines and nano adjuvants heighten the ability of antigen presentation, realizing super-additive synergistic effects. As summarized, these strategies of nano-biomaterials-mediated radio-immunotherapy have generated remarkable synergistic outcomes.

However, several obstacles, such as the unknown molecular mechanisms of immune pathways, the uncertainty of the side effects for nano-biomaterials-activated immunity, and the complexity of the interplay between nanomedicine and radio-immunotherapy, remain to be overcome. To promote the clinical translation of nano-biomaterials-assisted radio-immunotherapy, there are some critical issues to be addressed. (1) The interaction between nanoformulations and biological organs, tissues, or cells is still largely unknown due to the complexity of current nanodelivery technologies. In particular, much work is required to understand the interaction between a patient's systemic immunity and these complex nanodrugs. (2) Understanding the mechanism of nano-biomaterials-enhanced radio-immunotherapy. The development and metastasis of malignant tumors are the results of the interaction between the immune system and the body. An improved understanding of the mechanism of immunization would help to develop tumor immune-related nano-biomaterials. (3) Large-scale human tumor-derived sample analysis in animal models is lacking. Murine cell lines are frequently used for the majority of pre-clinical investigations in radio-immunotherapy. A few humanized murine models still struggle with problems like rejection, a subpar or nonexistent immune response to MHC-restricting antigens, and a challenging modeling procedure. (4) The biological safety of nano-biomaterials in the application of tumor radio-immunotherapy has drawn much attention. In addition to requiring high biocompatibility of nano-biomaterials themselves, safer ways of drug administration should also be selected. At present, intratumoural administration has attracted wide attention from researchers. It has been reported that intratumoural administration can increase drug availability in situ and increase body tolerance. Importantly, intratumoural administration also provides the advantage of access to lymph nodes with anti-tumor immune response [91]. Overall, the development of functionalized nano-biomaterials will make great contributions to radio-immunotherapy and provide the synergistic cancer immunotherapy for clinical translation.

## Data Availability

Yes.

## Abbreviations

RT: Radiotherapy
NK: Natural killer
APC: Antigen-presenting cells
PCa: Prostate cancer
TME: Tumor microenvironment
HLA-E: Human leukocyte antigen E
αPD-1: Anti-Programmed Death 1
αPD-L1: Anti-Programmed Death Ligand 1
αCTLA-4: Anti-Cytotoxic T-Lymphocyte Antigen 4
IDOi: Indoleamine 2,3-dioxygenase inhibitor
AC-NPs: Antigen capturing poly (lactic-co-glycolic acid) nanoparticles
WSP NPs: WO_2.9_-WSe_2_-PEG nanoparticles
^177^Lu: Lutecium-177
^177^Lu@Au NCs: Lutecium-177 on the metabolizable gold nanoclusters
LNP: Lipid nanomaterials
SLN: Solid lipid nanoparticles
Treg: Regulatory T cells
ROS: Reactive oxygen species
IDO: Indoleamine 2,3-dioxygenase
IDOi: IDO inhibitor
nMOF: Nanoscale metal–organic framework
CaCO_3_: Calcium carbonate
HIF: Hypoxia-inducible factor
ALG: Sodium alginate
Cat: Catalase
PLGA: Poly (lactic-co-glycolic) acid
MOF: Metal–organic framework
•OH: Hydroxyl radical
GSH: Glutathione
ROS: Reactive oxygen species
SM NPs: Si-Mn-based NPs
IFN: Interferon
STING: Stimulator of interferon genes
ATRi: ATR kinase inhibitors
HfO_2_: Hafnium oxide
ANPs: Astragalus polysaccharide nanoparticles
DOX: Doxorubicin
